# Cystic Fibrosis Bronchial Epithelial Cells Are Lipointoxicated by Membrane Palmitate Accumulation

**DOI:** 10.1371/journal.pone.0089044

**Published:** 2014-02-19

**Authors:** Laurie-Anne Payet, Linette Kadri, Sébastien Giraud, Caroline Norez, Jean Marc Berjeaud, Christophe Jayle, Sandra Mirval, Frédéric Becq, Clarisse Vandebrouck, Thierry Ferreira

**Affiliations:** 1 Signalisation et Transports Ioniques Membranaires, ERL CNRS 7368, Université de Poitiers, Poitiers, France; 2 Ecologie et Biologie des Interactions, UMR CNRS 7267, Université de Poitiers, Poitiers, France; 3 Service de Biochimie, CHU Poitiers, Poitiers, France; 4 Inserm U1082, Poitiers, France; 5 Faculté de Médecine et de Pharmacie Université de Poitiers, Poitiers, France; 6 Service de Chirurgie Cardiothoracique, CHU Poitiers, Poitiers, France; University of Colorado, Denver, United States of America

## Abstract

The *F508del-CFTR* mutation, responsible for Cystic Fibrosis (CF), leads to the retention of the protein in the endoplasmic reticulum (ER). The mistrafficking of this mutant form can be corrected by pharmacological chaperones, but these molecules showed limitations in clinical trials. We therefore hypothesized that important factors in CF patients may have not been considered in the *in vitro* assays. CF has also been associated with an altered lipid homeostasis, *i. e.* a decrease in polyunsaturated fatty acid levels in plasma and tissues. However, the precise fatty acyl content of membrane phospholipids from human CF bronchial epithelial cells had not been studied to date. Since the saturation level of phospholipids can modulate crucial membrane properties, with potential impacts on membrane protein folding/trafficking, we analyzed this parameter for freshly isolated bronchial epithelial cells from CF patients. Interestingly, we could show that Palmitate, a saturated fatty acid, accumulates within Phosphatidylcholine (PC) in CF freshly isolated cells, in a process that could result from hypoxia. The observed PC pattern can be recapitulated in the CFBE41o^−^ cell line by incubation with 100 µM Palmitate. At this concentration, Palmitate induces an ER stress, impacts calcium homeostasis and leads to a decrease in the activity of the corrected F508del-CFTR. Overall, these data suggest that bronchial epithelial cells are lipointoxicated by hypoxia-related Palmitate accumulation in CF patients. We propose that this phenomenon could be an important bottleneck for F508del-CFTR trafficking correction by pharmacological agents in clinical trials.

## Introduction

Cystic fibrosis (CF) is a genetic disease caused by mutations in the *CFTR* (CF Transmembrane conductance Regulator) gene, encoding a plasma-membrane chloride channel. Although this disease has been described for the first time in 1936 and 1938 by Franconi and Anderson, respectively, as the CF of the pancreas [1], [2], the corresponding gene was identified later in 1989 [3], [4]. More than 1,900 mutations of *CFTR* responsible for the disease have now been listed by the Cystic Fibrosis Genetic Analysis Consortium. The most common mutation worldwide, the deletion of phenylalanine at position 508 (*F508del-CFTR*), leads to the mistrafficking of the corresponding protein, resulting in its retention in the endoplasmic reticulum (ER) and its degradation by the ER-associated protein degradation (ERAD) pathway [5]–[7].

Since the *CFTR* gene discovery, many efforts have been concentrated on correcting the F508del-CFTR trafficking defect. Some molecules were found to act as pharmacological chaperones or as proteostasis modulators during the F508del-CFTR folding process in the ER, restoring its trafficking to the plasma membrane. Among these correctors, 4-PBA (sodium 4-phenylbutyrate) [8], CPX (8-cyclopentyl-1,3-dipropylxanthine) [8], VX809 [9] and miglustat [10] have been tested in clinical trials but, until now, they appeared to be less efficient *in vivo* than *in vitro* [11]–[14]. Our study falls within this context. We hypothesized that important factors in CF patients may have not been taken into consideration in the *in vitro* assays, therefore accounting for the clinical trial failure.

Long before the *CFTR* gene discovery, CF had already been related to an altered lipid homeostasis. Indeed, the disease had been associated since the early 60′s to low concentrations of polyunsaturated fatty acids (PUFA), such as linoleic acid (C18∶2, [15]) and docosahexaenoic acid (C22∶6, [16]–[18]), in CF patient plasma and tissues. Fatty acids (FA) are the components of phospholipids (PL) and the species containing PUFA were also shown to be decreased in the plasma of CF patients, particularly phosphatidylcholine (PC) containing PUFA [19].

Nevertheless, to the best of our knowledge, there is no study that clearly determined the precise fatty acyl content of membrane phospholipids in bronchial epithelial cells from CF patients. More specifically, several studies in different cellular models demonstrated that the ratio of Unsaturated (UFA) versus Saturated Fatty Acid (SFA) phospholipid-containing species regulate crucial membrane biophysical properties and therefore control the functionality of intracellular organelles [20], [21]. As an example, in previous studies using a simple eukaryotic model, the yeast *Saccharomyces cerevisiae*, we could show that SFA accumulation within PL decreases membrane fluidity [22], and induces the accumulation of misfolded proteins within the ER, which results in the induction of an ER-based response referred to as the Unfolded Protein Response (UPR). When sustained, UPR induction results in an ER-stress, which can ultimately lead to cell death by apoptosis [21]. Vesicular budding at the *Trans* Golgi network [23] and then membrane protein trafficking latter in the secretory pathway are also altered under SFA accumulation [20]. In other words, increased SFA amounts within PL impact the secretory pathway as a whole [23]. If such a process occurred in CF cells *in vivo* (*i. e.* SFA-containing PL increasing at the expense of UFA-containing species), it could alter F508del-CFTR trafficking correction by pharmacological means.

In the present study, we therefore focused on the fatty acyl content of membrane PL in bronchial epithelial cells from CF patients. We first show that Palmitate (C16∶0) accumulates within PC from CF patient cells but not in the corresponding CFBE41o^−^ cell line. Then, this CF cellular model was optimized to mimic the PL content encountered *in vivo* by adding exogenous Palmitate to the culture medium. At a concentration of 100 µM Palmitate, which results in a PC pattern very similar to the one observed in freshly isolated CF patient cells, CFBE41o^−^ cells do not fall prey to apoptosis, but they appear to experience an ER stress. Moreover, extracellular Ca^2+^ influx and F508del-CFTR correction by pharmacological agents are significantly altered by Palmitate accumulation within PL.

## Materials and Methods

### Ethics Statement

The study was approved by our local institutional ethics committee (Comité de Protection des Personnes (CPP) Ouest III). All participants provided their written informed consent to participate in this study, and the ethics committee approved this consent procedure.

### Freshly Isolated Bronchial Epithelial Cells

Human lung tissue was obtained from seventeen individuals: five non-CF males with a mean age of 61 yrs, two non-CF females with a mean age of 67 yrs, four *F508del/F508del-CFTR* males with a mean age of 27 yrs, and six *F508del/F508del-CFTR* females with a mean age of 28 yrs. Following lobectomy, lung samples were quickly dissected. After removal of connective tissues, cartilage and smooth muscle tissues, bronchial tubes were washed at least three times with phosphate-buffered saline (PBS, Gibco) and cut out in small segments. Epithelial cells were dissociated using enzymatic isolation for 24 h at 4°C with 0.1% protease and 0.01% deoxyribonuclease in a decontamination medium composed by Dulbecco’s modified Eagle’s medium-Ham’s F-12 medium (DMEM/Ham’s F12, Gibco) supplemented with penicillin/streptomycin 1 mg/mL (Sigma-Aldrich), amphotericin B 100 µg/mL (Sigma-Aldrich), gentamycin 0.5 mg/mL (Sigma-Aldrich) for non-CF samples and the same medium supplemented with ceftazidin 500 µg/mL (Sigma-Aldrich) and ticarcilin 500 µg/mL (Sigma-Aldrich) for CF samples. After neutralization by 10% fetal bovine serum (FBS), cells were centrifuged at 600 rpm for 6 min. Freshly isolated bronchial epithelial cells were washed three times with PBS and counted using a Malassez cell.

### Cell Culture

The human CF and non-CF bronchial epithelial CFBE41o^−^ (homozygote *F508del-CFTR*) and 16HBE14o^−^ (wild type CFTR) cell lines were grown at 37°C in 5% CO_2_ and 95% O_2_ on Corning**®** CellBIND**®** surface cell culture flasks (Sigma-Aldrich), in a minimum essential medium containing L-glutamine (MEM, 31095, Gibco) and supplemented with plasmocin (5 µg/mL) and 10% serum (FBS or horse serum, HS). Frozen cells in FBS were thawed in a FBS-containing medium [24]. Two days after the thawing, cells are incubated in a medium containing 5% FBS and 5% HS during 48 h, before switching in a medium containing only HS. Culture medium was renewed at 2-day intervals. For hypoxia experiments, cells were grown into a hypoxia chamber at 37°C in 5% CO_2_ and 95% N_2_ during 48 h.

### Fatty Acid (FA) Preparation and Cell Treatment

Palmitate preparation was described by Karaskov and colleagues [25]. Briefly, 10 mM Palmitate (Sigma-Aldrich) stocks were prepared in filtered 0.1 M NaOH and heated at 70°C. A 5% (w/v) FA-free BSA (Sigma-Aldrich) solution was prepared in double distilled H_2_O and filtered. A five time more concentrated FFA/BSA solution (for example, 5 mM FFA/5% BSA) was prepared by complexing an appropriate amount of FFA to 5% BSA in a 60°C water bath. The above solution was then cooled to room temperature and diluted 1∶5 in culture medium without HS to the appropriate final concentration (for example 1 mM FFA/1% BSA) for cells incubation during 16 h.

### Lipid Extraction, Phospholipid Purification and Mass Spectrometry Analyses

Lipid extracts were prepared from approximately 10^5^ cells, grown as indicated. Cells were harvested, washed with PBS and the cell pellets were resuspended in 1 mL of water and transferred into glass tubes with 500 µL of glass beads (diameter 0.3–0.4 mm; Sigma-Aldrich). Lipids were extracted using chloroform/methanol (2∶1, v/v) and shaking on an orbital shaker (IKA® VXR basic Vibrax®, Sigma-Aldrich) at 1,500 rpm during at least 1 h. The protocol used was described by Folch et *al.* [26]. The final organic phase was evaporated and dissolved in 100 µL chloroform/methanol/H_2_O (16∶16:5, v/v/v) for analysis of total lipids by mass spectrometry or 100 µL dichloromethane for PL purification on a silica column (Bond ELUT-SI 100 mg 1 mL, Agilent Technologies).

Before PL purification, the silica column was washed with 3 mL methanol and 2 mL dichloromethane. Lipid samples were loaded on the top of the column. Non polar lipids were eluted by addition of 2 mL dichloromethane, and glycolipids with 3 mL acetone. PL were then eluted by 2 mL chloroform/methanol/H_2_O (50∶45:5, v/v/v).

To analyze PC species by ESI-MS (Electrospray Ionization Mass Spectrometry) in the positive ion mode, total lipid extracts or purified PL were evaporated and reconstituted in 1% (v/v) formic acid. The molecular profile of PC species was specifically obtained by scanning for the positive ion precursors of m/z 184, characteristic of choline phosphate. Due to material limitations, particularly with freshly isolated bronchial epithelial cells, samples were not analyzed in the negative ion mode in this study.

### RT-qPCR

RNA extraction was from 10^7^ cells using a commercial kit (RNeasy Mini Kit, Qiagen). Reverse transcription-PCR (RT-PCR) was done with SuperScript II (Invitrogen) using the procedure supplied by the manufacturer. Hypoxia-inducible factor 1α (*HIF1*) expression was assessed relative to Glyceraldehyde 3-phosphate dehydrogenase (*GAPDH*) expression by real-time quantitative PCR (RT-qPCR) with the GeneAmp 9500 Sequence Detection System and SYBR Green chemistry (Applied Biosystems). The RT-qPCR primers used were: *GAPDH*, 5′-TGCACCACCAACTGCTTAGC-3′ and 5′-GGCATGGACTGTGGTCATGAG-3′; and *HIF-1α*, 5′-CATTACCCACCGCTGAAACG-3′ and 5′-TTCACTGGGACTATTAGGCTC-3′ [27].

### Apoptosis Quantification

Palmitate-induced apoptosis was monitored using the cell death detection kit ELISA^PLUS^ (Roche). CFBE41o^−^ cells were seeded in 96-well plates (10,000 cells per well). 4 h later, cells were incubated with BSA-conjugated Palmitate in a serum-free medium during 16 h. After the treatment, cells were lysed and apoptosis was measured according to the manufacturer’s instructions by quantifying cytoplasmic oligonucleosomes which are indicative of apoptosis-associated DNA degradation.

### Western Blot

Cells grown on a 60 mm dish were lysed by scraping after addition of 100 µL lysis buffer: Tris 10 mM pH 7.5, NP40 1% (v/v), DOC 0.5% (w/v) supplemented with a cocktail of protease inhibitors (Roche, 2%, v/v) and the phosphatase inhibitor cocktail 3 (Sigma-Aldrich, 1%, v/v). Total proteins were quantified using the bicinchoninic acid (BCA) protein assay (Sigma-Aldrich). 40 µg of total protein extract were resolved by SDS-PAGE, transferred to nitrocellulose membranes and immunoblotted with antibodies directed against the phosphorylated eukaryotic translation initiation factor 2α (P-eIF2α, Cell Signaling, 1∶200), eIF2α (Cell Signaling; no. 9722, 1∶500) and β tubulin (Santa Cruz Biotechnology, 1∶2000). After incubation with the secondary antibody (donkey anti-rabbit IgG, or sheep anti-mouse IgG, GE Healthcare, 1∶5000) conjugated to horseradish peroxidase (HRP), the bands were detected with the Immobilon Western Chemiluminescent HRP substrate (Merck Millipore, USA) or with ECL Select™ (GE Healthcare). Immunoblots were scanned and quantified by densitometry using ImageJ software.

### Calcium Signal Recording

CFBE41o^−^ cells, grown till 80% of confluence on a glass slide, were incubated or not with Palmitate during 16 h. Then, they were loaded with 3 µM Fluo-4 acetoxymethyl (AM) ester (FluoProbes®) for 20 min at room temperature in a 2Ca^2+^ buffer solution (NaCl 118.4 mM, KCl 4.7 mM, CaCl_2_ 2.5 mM, KH_2_PO_4_ 1.2 mM, MgSO_4_ 1.2 mM, NaHCO_3_ 25 mM, Glucose 11.7 mM, pH 7.4). Global Ca^2+^ mobilization was induced by the addition of 100 µM ATP. For ER Ca^2+^ store depletion, induced by the addition of 10 µM thapsigargin, 2Ca^2+^ buffer solution was replaced by 0Ca^2+^ buffer solution (NaCl 118.4 mM, KCl 4.7 mM, KH_2_PO_4_ 1.2 mM, MgSO_4_ 1.2 mM, NaHCO_3_ 25 mM, Glucose 11.7 mM, pH 7.4) just before recording. Ca^2+^ activity was assessed using a Zeiss Axio observer Z1 inverted microscope and the Carl Zeiss AxioVision Release 4.8.2 software. Fluorescence intensity of selected cells was measured every 2 sec during 5 min. Intensity profiles were normalized by dividing the fluorescence intensity of each pixel (F) by the average resting value before stimulation (F0) to generate an (F − F0/F0) image. All experiments were performed on a minimum of two different cell passages.

### Iodide Efflux

The activity of swelling-dependent (Cl_swell_), Ca^2+^-dependent (Cl_Ca_) and CFTR Cl^−^ channels was assessed on the CFBE41o^−^ cell population by the iodide (^125^I^−^) efflux technique as already described [28]. Curves were constructed by plotting the rate of ^125^I^−^ versus time. All comparisons were based on maximal values for the time-dependent rates (k = peak rates, min^−1^), excluding the points used to establish the baseline (k_peak_ – k_basal_, min^−1^). All chemicals were from Sigma-Aldrich, except for miglustat (Toronto Research Chemicals, Canada) diluted in water, forskoline and genistein (LC Laboratories, USA) diluted in DMSO.

## Results

### Palmitate Accumulates within PC of Bronchial Epithelial Cells from CF Individuals, but this Accumulation is Not Directly Related to the F508del-CFTR Mutation

**Figure 1:**
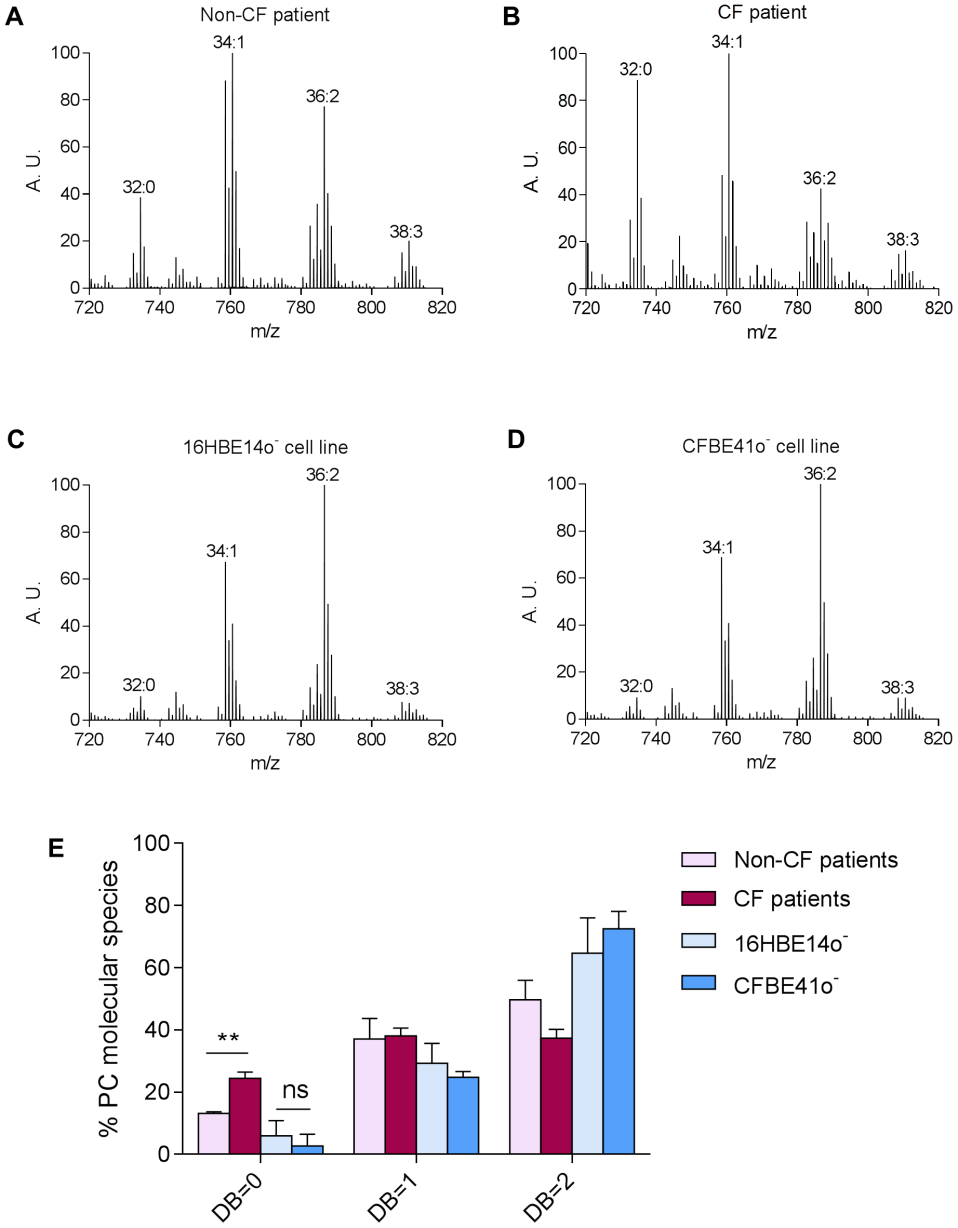
PC profiles of CF and non-CF cells. Characteristic PC profiles (positive ion mode) obtained from bronchial epithelial cells of A) non-CF or B) CF patient lungs, and from C) non-CF and D) CF bronchial epithelial cell lines. The total carbon chain length (x) and number of carbon-carbon double bounds (y) of the main PC molecular species are indicated (x:y). A. U., arbitrary unit. E) The relative percentage of saturated (DB = 0) versus monounsaturated (DB = 1) and diunsaturated (DB = 2) species were calculated from the mass spectra. Values are means ± SEM of at least three independent measurements. The *P*value was calculated by a two-tailed *t* test, using Graphpad Prism 6 software. **: p<0.01. ns: no significant difference. Schematic representations of the major PC species are included.

Lipids extracted from bronchial epithelial cells of non-CF and CF patient lung samples were analyzed by mass spectrometry. Examples of characteristic positive ion mode spectra are displayed in Figure 1A (non-CF patient) and 1B (CF patient). For peak assignment, ions were subjected to ion scan analyses as described previously [29]. In this figure, the main peaks corresponding to molecular species of PC are indicated. The detailed composition of the various PC species in each sample is displayed in Table S1 in File S1. Interestingly, the most prominent differences between non-CF and CF patients were not observed for PC species bearing PUFA chains (*e. g.* PC(38∶3); compare Fig. 1A and 1B). Moreover, PC species bearing longer polyunsaturates were hardly detectable in freshly isolated bronchial epithelial cells (not-shown). Rather, the obtained PC profiles revealed that PC(32∶0), a PC species bearing two saturated (*i. e.* Palmitate C16∶0) side chains, was very abundant in CF patient (2-fold increase; Fig. 1B), as compared to non-CF patient cells (Fig. 1A and Table S1 in File S1). This shift towards more saturated lipids can be visualized by representing the PC Double-Bond (DB) index (Fig. 1E). As shown, in CF patients, this shift is manifested by a dramatic increase of PC with two saturated acyl chains (*i.e.* total number of double bonds in both acyl chains is zero; DB = 0) at the expense of PC with two monounsaturated acyl chains (DB = 2). As shown on Figure 1A, the most represented DB = 2 species is PC(36∶2), which corresponds to a PC species bearing two Oleate chains (C18∶1). To check the link between the *F508del-CFTR* mutation and Palmitate accumulation within PC, the same experiment was performed on non-CF (Fig. 1C) and CF (Fig. 1D) bronchial epithelial cell lines. Since the fatty acid composition of cells in culture is reflective of what is present in the cell culture media, the cell lines were grown in horse serum which displays a fatty acid profile that matches at best the one encountered in the human plasma [24]. Surprisingly, the two cell lines displayed the exact same PC profile, and no Palmitate accumulation within PC was observed in the presence of the *F508del-CFTR* mutation (compare Fig. 1C and 1D; Table S1 in File S1). Therefore, despite the fact that CFBE41o^−^ cells are bronchial epithelial cells and that they are homozygous for the *F508del-CFTR* mutation as the cells collected from patients, they do not reflect the PC pattern observed in freshly isolated cells.

### The Palmitate Accumulation within PC Could be the Result of Hypoxia

**Figure 2:**
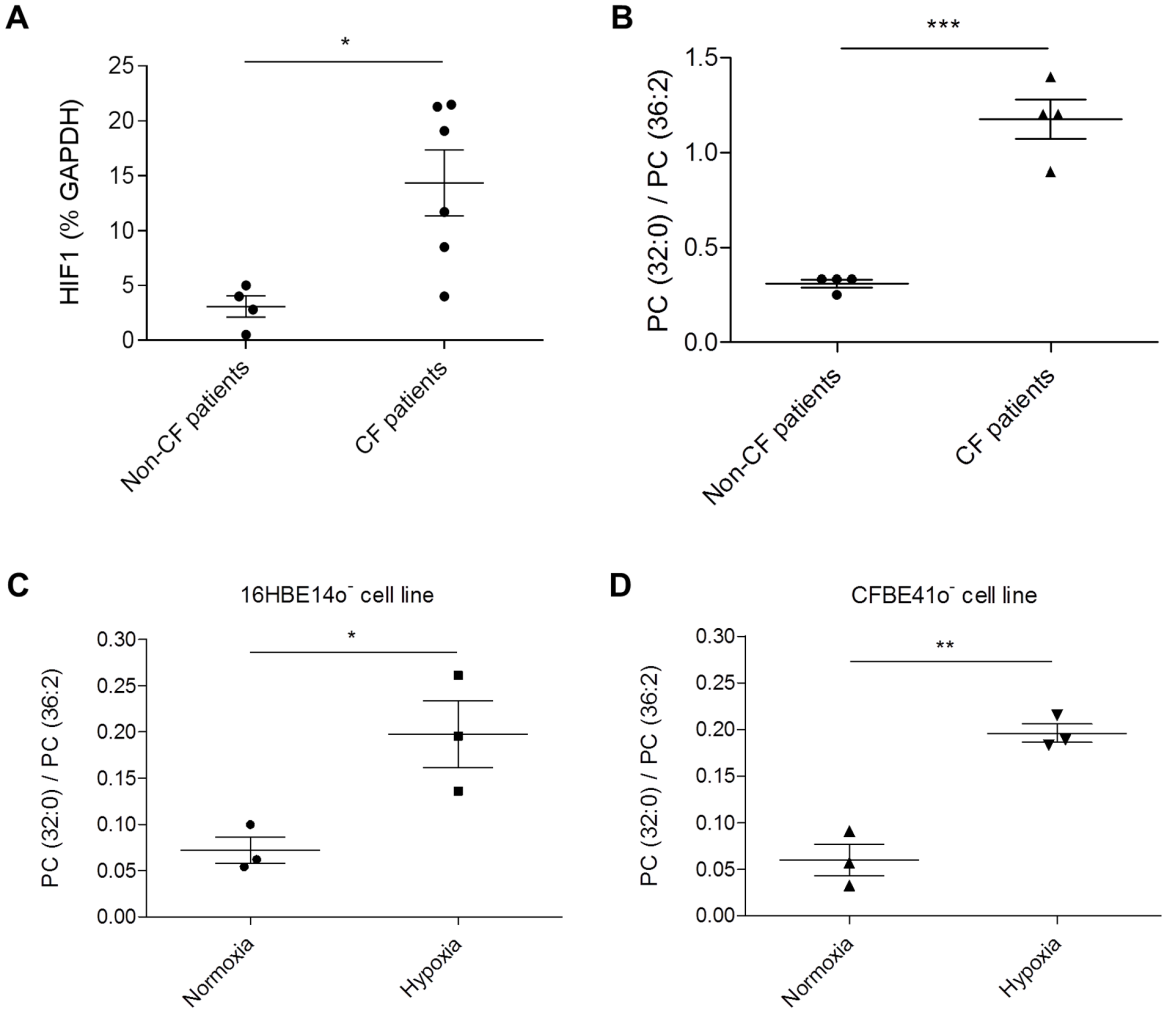
Relationship between PC saturation rate and hypoxia. A) *HIF1α* expression in non CF and CF patient bronchial epithelial cells, expressed as a percentage of *GAPDH*, used as a standard. Each point represents the mean of three determinations for one patient sample. Saturation rate (SR) of B) patient cells, C) non-CF and D) CF bronchial epithelial cell lines after incubation or not into a hypoxia chamber without oxygen during 48 hours. SR corresponds to the ratio PC(32∶0)/PC(36∶2), calculated from the mass spectra. Each point represents the mean of three determinations for one patient or one passage. Means ± SEM are indicated. The *P*value was calculated by a two-tailed *t* test, using Graphpad Prism 6 software. *: p<0.05. **: p<0.01. ***: p<0.001.

This Palmitate accumulation within PC in CF patient cells could be of physiological relevance since we have already shown that saturated PL impact the secretory pathway by altering membrane properties [22] and, as a consequence, the trafficking of plasma-membrane proteins to their final destination [20], [21], [23]. In order to improve the CFBE41o^−^ model in terms of its lipid composition, we tried to figure out what could be the origin of this Palmitate accumulation within PC in the lungs of CF individuals. Based on the literature and physiological observations, we hypothesized that hypoxia could be the link between the disease and the observed Palmitate accumulation. Indeed, in CF, respiratory airways are obstructed by a thick layer of viscous mucus, which deprives the epithelial cells of oxygen [30]. Furthermore, enzymes that desaturate FA (*i. e.* which catalyze the conversion of a single bound between two carbon atoms (C-C) to a double bound (C = C) in fatty acyl chains) are oxygen-dependent [31]. In the case of a decrease in oxygen availability, they are not functional anymore and SFA tend to accumulate within the cells. Relevantly, this property was fruitfully used in yeast to provoke SFA-accumulation within PL [32]. To test this hypothesis, the expression of the hypoxia-inducible factor 1α (*HIF1α*), a transcription factor activated by the decrease in oxygen availability [33], was determined in bronchial epithelial cells from CF and non-CF patient lungs (Fig. 2A). Interestingly, *HIF1α* expression levels were significantly higher in CF patient cells compared to the non-CF ones, therefore reinforcing our original hypothesis.

In a next step, we tried to recreate the hypoxia conditions *in vitro*, by growing 16HBE14o^−^ and CFBE41o^−^ cells in a hypoxia chamber, where oxygen can be totally replaced by nitrogen (Fig. 2C and 2D). After 48 h under these conditions, lipids were extracted from the cells and analyzed by mass spectrometry (detailed PC compositions are given in Table S2 in File S1). To get a more quantitative vision, the saturation rate, corresponding to the ratio of the characteristic PC(32∶0) over PC(36∶2) species (Fig. 1) was calculated from the mass spectra obtained. The analyses revealed that the saturation rate was 4-fold higher in hypoxia than under normoxia for both cell lines. In other words, oxygen scarcity resulted in the accumulation of Palmitate within PC species. However, the saturation rates obtained under hypoxic conditions were not as spectacular as the ones observed *in vivo* (Fig. 2B). These discrepancies are likely related to the following facts/observations: *i*) the original PC(32∶0) levels in both cell lines grown under normoxia were very low, even when compared to the profiles obtained from non-CF patient cells (Fig. 1E), *ii*) a 48 h incubation in the hypoxia chamber may not fully mimic the chronic hypoxia that CF patients are subjected to, and iii) complete oxygen deprivation in the hypoxia chamber reduced the cell division rate (our unpublished data), with likely impacts on PC turn-over and, as a consequence, SFA-accumulation rates within this phospholipid species.

Altogether, if these experiments clearly demonstrated that hypoxia could account for the Palmitate accumulation observed in patient lungs, the use of a hypoxia chamber to mimic the CF PC pattern in the CFBE41o^−^ cell line showed important limitations.

### Exogenous Palmitate Intake can be used to Mimic the PC Pattern Observed in Freshly Isolated CF Bronchial Epithelial Cells

**Figure 3:**
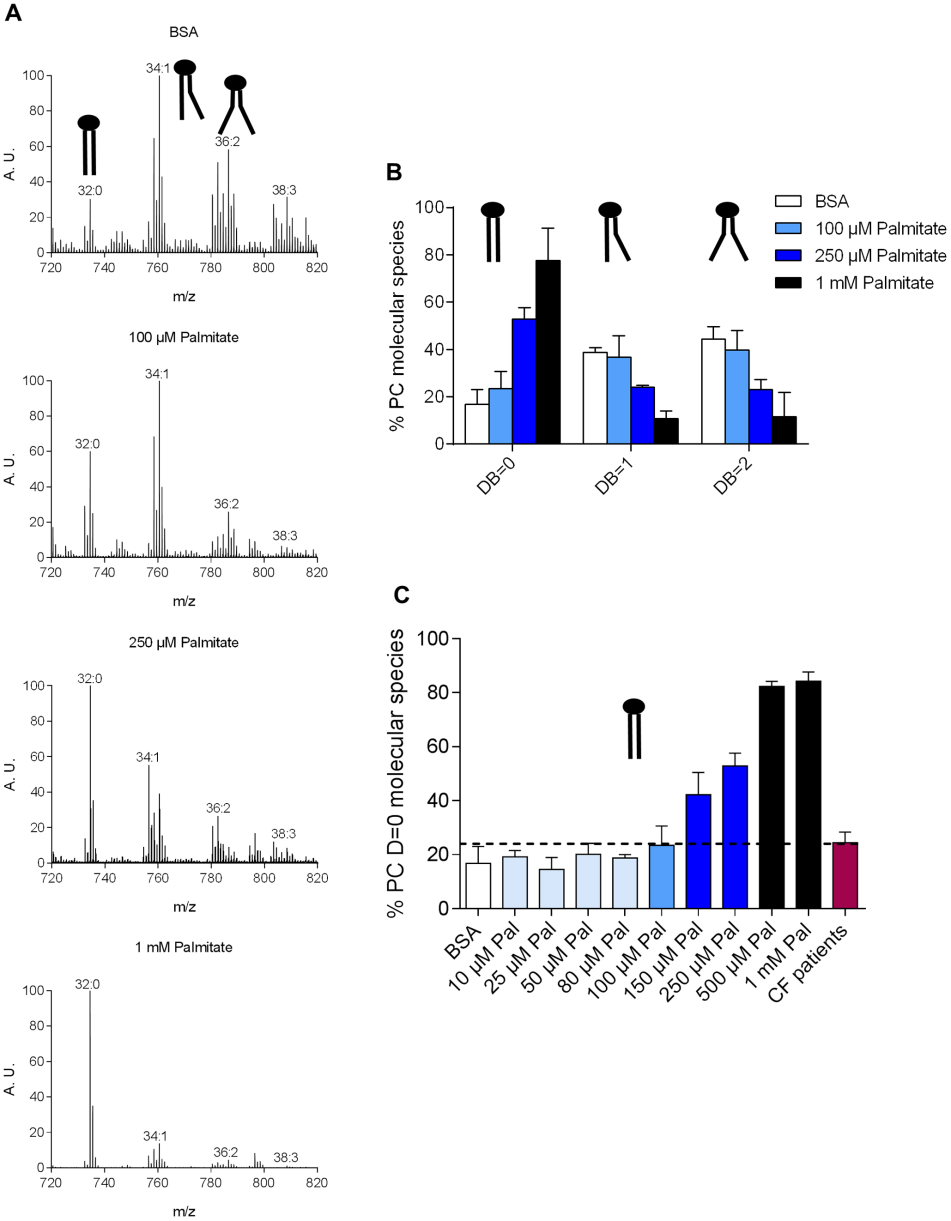
Effects of exogenous Palmitate intake on the fatty acyl content of PC. A) Positive ion mass spectra specific for molecular species of PC extracted from CFBE41o^−^ cells after incubation with 1% BSA, 100 µM Palmitate, 250 µM Palmitate or 1 mM Palmitate during 16 h. The total carbon chain length (x) and number of carbon-carbon double bounds (y) of the main PC molecular species (x:y) are indicated. A. U., arbitrary unit. B) Relative percentage of saturated (DB = 0) versus monounsaturated (DB = 1) and diunsaturated (DB = 2) species of CFBE41o^−^ after incubation with Palmitate at the indicated concentrations, were calculated from the mass spectra. C) Relative percentage of saturated PC species of CFBE41o^−^ cells after incubation with Palmitate concentrations ranged from 0 to 1 mM. Values are means ± SD of at least three independent measurements. Schematic representations of the major PC species are included.

In order to evaluate the impacts of Palmitate accumulation within PC in CF cells, we therefore developed an alternative strategy to improve Palmitate accumulation within the CFBE41o^−^ cells: this consisted in supplying the culture medium with an exogenous Palmitate source. This approach proved to be efficient with different cellular models, including mammalian β-cells and hepatocytes [25], [34]. BSA-conjugated Palmitate was added to the CFBE41o^−^ cell cultures without serum at a final concentration of 1 mM, for 16 h. Lipids were extracted and purified PL were analyzed by mass spectrometry. As shown on Fig. 3A, CFBE41o^−^ cells incubated with 1% BSA displayed a non CF-like PC profile (Fig. 1A). In contrast, when incubated with 1% BSA-1 mM Palmitate, PC(32∶0) became the major, if not the only, PC species in CFBE41o^−^ cells. These data indicate that exogenous Palmitate can be efficiently incorporated within PC, but that a concentration of 1 mM Palmitate is too high to reproduce the PC pattern observed in freshly isolated cells.

Therefore, various BSA-conjugated Palmitate concentrations, ranging from 0 to 1 mM Palmitate, were tested. The characteristic PC profiles obtained with 1% BSA, 100 µM, 250 µM and 1 mM Palmitate (Fig. 3A), showed that PC(32∶0) species levels increased with Palmitate concentration in the culture medium (detailed PC compositions are given in Table S3 in File S1). More generally, increasing the exogenous Palmitate supply resulted in an increase of the saturated species (Fig. 3B, DB = 0), at the expense of the mono- and diunsaturated species (Fig. 3B; DB = 1 and DB = 2, respectively). To find the concentration which could mimic the CF PC pattern at best, we compared the DB = 0 obtained for each tested concentration with the one obtained for CF patients (Fig. 3C). 100 µM Palmitate resulted in a PC profile matching at best the CF PC pattern. Therefore, this concentration was retained for further experiments.

### High PC Saturation Rates Induce Apoptosis in Bronchial Epithelial Cells

**Figure 4:**
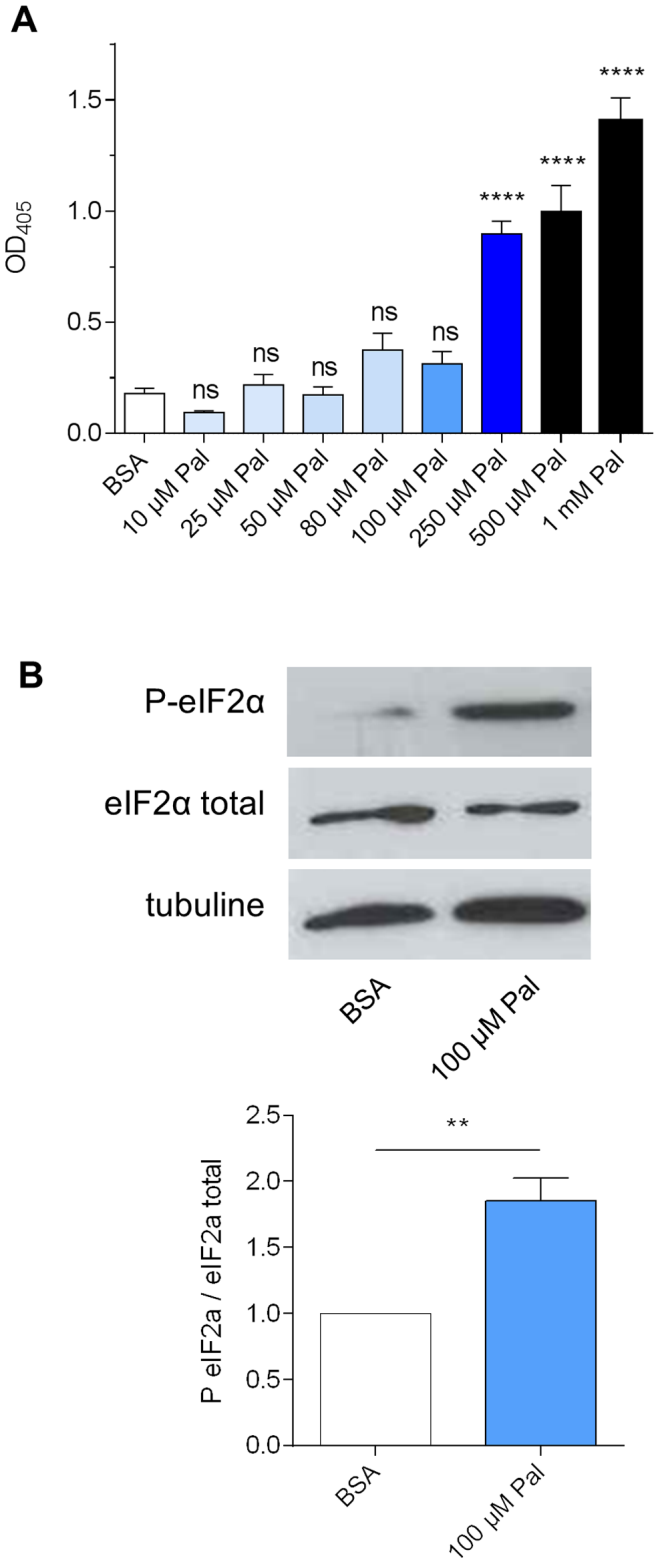
Effects of fatty acyl content of PC on apoptosis induction. A) Apoptosis quantification in CFBE41o^−^ cells after incubation with Palmitate, during 16 h, using the cell death detection ELISA^PLUS^ kit (Roche). The *P*value was calculated by a One-way ANOVA test, using Graphpad Prism 6 software. ****: P<0.0001; ns, no significant difference compared to the control (BSA). B) Phosphorylation rate of eIF2α, an ER stress marker. Top: representative western blot of total protein extracts from CFBE41o^−^ cells after treatment with BSA (control) or 100 µM Palmitate. Bottom: quantification of eIF2α phosphorylation as ratios of P-eIF2α versus total eIF2α, assuming a value of 1 for the ratio determined from the control. All values, obtained from five independent experiments, were normalized to a loading control (β tubulin). Means ± SEM are represented. The *P*value was calculated by a two-tailed *t* test, using Graphpad Prism 6 software. **: p<0.01.

In β cells, Palmitate accumulation, obtained by the addition of 500 µM Palmitate in the culture medium, has been shown to induce apoptosis [35]. We therefore evaluated whether this could be also the case for bronchial epithelial cells under Palmitate treatment. To this aim, after incubation of CFBE41o^−^ cells with the same range of Palmitate concentrations used for the PC profile study, induction of apoptosis was monitored using a commercial kit that measures the accumulation of cytoplasmic histone-associated DNA fragments, a characteristic trademark of apoptosis [36]. As shown on Fig. 4A, apoptosis was significantly induced in this cell type from a Palmitate concentration of 250 µM.

Interestingly, the Palmitate concentration of 100 µM, which results in a PC pattern similar to the one encountered in CF patients, was not associated with a clear-cut induction of apoptosis in CFBE41o^−^ cells (Fig. 4A). In yeast and in β cells, apoptosis induction by SFA, and particularly Palmitate, is paralleled by the induction of an ER-based stress response, namely the UPR [21], [37]. We therefore questioned if, at this Palmitate concentration, CFBE41o^−^ cells could somehow experience such an ER-stress. In mammalian cells, UPR is composed of three independent pathways, each regulated by different proteins localized in the ER membrane: IRE1 (inositol requiring 1), ATF6 (activating transcription factor 6) and PERK (protein kinase like ER kinase). The PERK pathway is usually targeted for the study of SFA toxicity in β-cells [25], [35], [37], [38]. PERK, after activation by an ER stress, phosphorylates the α-subunit of the eukaryotic initiation factor 2 (eIF2α). Thus, the phosphorylation rate of eIF2α can be used as a marker of the SFA-induced ER stress. As shown on Fig. 4B, incubation of CFBE41o^−^ cells with 100 µM Palmitate resulted in a slight but significant increase of the phosphorylation rate of eIF2α, as compared to the control (BSA), suggesting that, at this Palmitate concentration, the PERK pathway is already induced.

Taken together, these data indicate that CFBE41o^−^ cells treated with 100 µM Palmitate to mimic the PC pattern observed in freshly isolated patient cells do not fall prey to apoptosis. However, they are experiencing an ER-stress.

### Effects of Exogenous Palmitate Intake on Calcium Homeostasis

SFA, and particularly Palmitate, are known to alter Ca^2+^ homeostasis in β cells and particularly ER Ca^2+^-stores. Even if data concerning this aspect are conflicting [25], [35], Cunha and collaborators showed that Palmitate-induced ER stress triggers the depletion of ER Ca^2+^ stores by the reducing ER Ca^2+^ uptake [35]. These observations were in agreement with the data from Li *et al.* who demonstrated that SFA, by increasing membrane order, inhibited the activity of the SERCA pump *in vitro*, which is responsible for the uptake of Ca^2+^ from the cytosol to the ER lumen [39].

**Figure 5:**
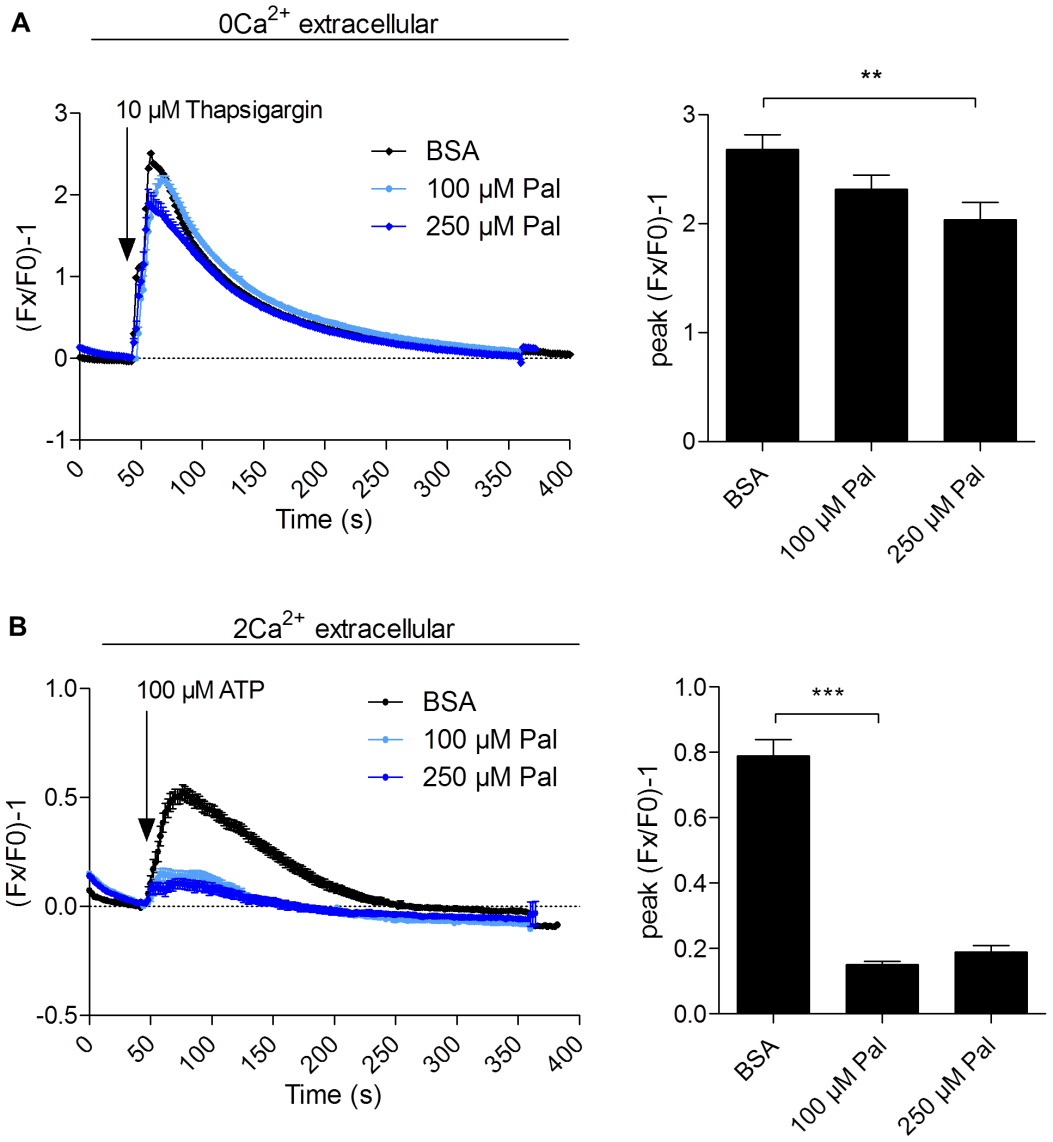
Effects of fatty acyl content on Ca^2+^ activity. Functional characterization of Ca^2+^ activity in CFBE41o^−^ cells after incubation with Palmitate, using the fluorescent Ca^2+^ probe Fluo4-AM. A) Representative traces of ER Ca^2+^ store depletion induced by 10 µM thapsigargin in the absence of extracellular Ca^2+^ (left) and corresponding histograms showing peak intensity (right). B) Representative traces of global Ca^2+^ mobilization induced by 100 µM ATP in the presence of extracellular Ca^2+^ (left) and corresponding histograms showing peak intensity (right). The experiments were performed on at least 60 cells from a minimum of 2 different passages. Values are means ± SEM. The *P*value was calculated by a two-tailed *t* test, using Graphpad Prism 6 software. **: p<0.01. ***: p<0.001.

In this context, we investigated the impacts of Palmitate accumulation within PL on Ca^2+^ homeostasis in CFBE41o^−^ cells (Fig. 5). In agreement with previous observations, ER Ca^2+^ stores were significantly depleted after treatment with 250 µM Palmitate (Fig. 5A). However, if a decrease in the amounts of Ca^2+^ released from the ER could also be observed with 100 µM Palmitate, this result was not significant as compared to the control (Fig. 5A). The most significant effects at this Palmitate concentration could be observed on global Ca^2+^ mobilization, which results from both mobilization of ER Ca^2+^ stores and Ca^2+^ influx from the extracellular medium (Fig. 5B). These results indicate that Ca^2+^ homeostasis is affected by 100 µM Palmitate treatments of CFBE41o^−^ cells, the main target of this alteration being the extracellular Ca^2+^ uptake by plasma membrane Ca^2+^ channels.

### Effects of Exogenous Palmitate Intake on F508del-CFTR Correction by Pharmacological Agents

**Figure 6:**
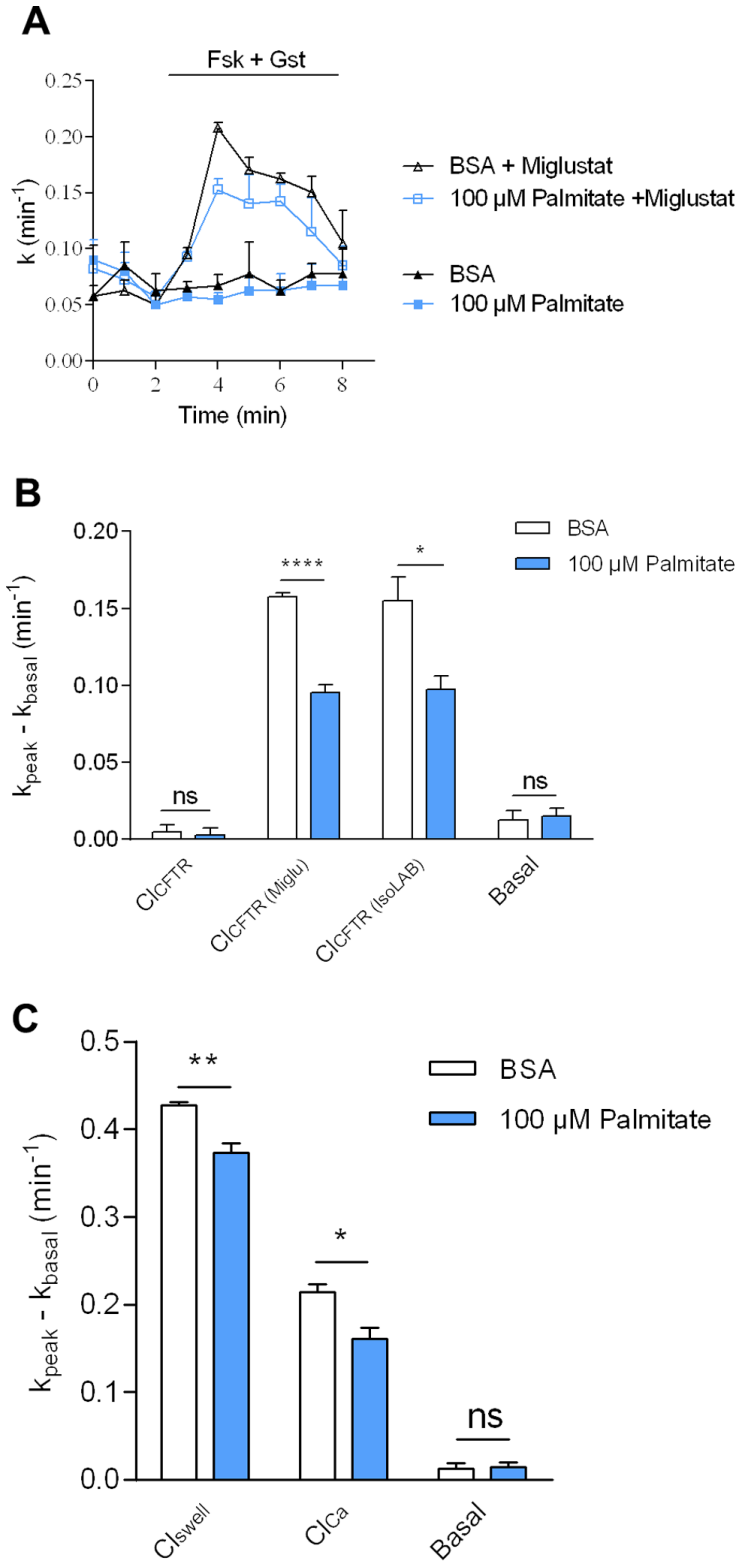
Effects of fatty acyl content of PC on Cl^−^ channels activity. A) Representative traces of iodide efflux curves obtained after stimulation of the F508del-CFTR activity by Forskoline (Fsk, 10 µM)+Genistein (Gst, 30 µM) in CFBE41o^−^ cells incubated or not with 100 µM Palmitate, and treated with 100 µM miglustat during 2 h. B) Histograms show the mean relative rate of F508del-CFTR activity corrected or not by miglustat (*n* = 4) or isoLAB (*n* = 4) after treatment with Palmitate. C) Histograms show the mean relative rate of Cl_swell_ after its stimulation by an osmotic shock, and Cl_Ca_ after its stimulation by the calcium ionophore A23187 (1 µM) in CFBE41o^−^ cells treated or not with Palmitate (*n* = 4). Means ± SEM are indicated. The *P*value was calculated by a two-tailed *t* test, using Graphpad Prism 6 software. *: p<0.05. **: p<0.01. ****: p<0.0001. ns: no significant difference compared to the control (BSA).

The fact that CFBE41o^−^ cells experienced an ER-stress under treatment with 100 µM Palmitate suggested that, at this concentration, intracellular organelle integrity and function might be somehow affected. Indeed, SFA accumulation with PL have been shown to reduce membrane fluidity, increase lipid packing and therefore impact several biophysical processes required for protein biogenesis and trafficking, such as folding [22] and vesicular budding [23]. In CF, the F508del-CFTR is retained in the ER before its degradation by the ERAD machinery. Nevertheless, its trafficking can be corrected by pharmacological agents, such as miglustat [10], which have been shown to be less efficient in preliminary clinical trials [11]–[14]. We therefore hypothesized that Palmitate accumulation within the PC of CF patient cells could impact F508del-CFTR trafficking correction. To test this hypothesis, we evaluated the activity of the F508del-CFTR channel by the iodide efflux technique, after incubation of CFBE41o^−^ cells with 100 µM Palmitate and a treatment with miglustat or isoLAB, another F508del-CFTR trafficking corrector [40], to restore F508del-CFTR trafficking. Fig. 6A shows a representative iodide efflux trace obtained for the study of the F508del-CFTR activity. Without treatment with correctors, F508del-CFTR cannot reach the plasma membrane and the relevant iodide efflux is close to zero. After treatment with miglustat, iodide efflux was restored but was 35% lower when cells were incubated with Palmitate (Fig. 6B). The same decrease in F508del-CFTR correction after Palmitate treatment was observed with isoLAB, (Fig. 6B). As plasma membrane Ca^2+^ intake was decreased under 100 µM Palmitate treatment (Fig. 5B), we wondered whether this Palmitate effect could be generalized to other membrane-embedded proteins than corrected F508del-CFTR or not. In this aim, we also tested the activity of two others chloride channels, the Cl_Ca_ channel after its stimulation by the calcium ionophore A23187, and the Cl_swell_ channel which is sensitive to cell swelling after activation by an osmotic shock (Fig. 6C). Such as corrected F508del-CFTR, iodide efflux was around 30% and 15% decreased for Cl_Ca_ and Cl_swell_ channels, respectively. These results suggest that Palmitate has global effects on plasma membrane chloride channels.

## Discussion

The most common mutation responsible for CF, *F508del-CFTR*, results in a misfolded protein that cannot reach its final destination, the plasma membrane. F508del-CFTR is retained in the ER before its degradation by the ERAD machinery [5]–[7]. However, the trafficking to the plasma membrane of this misfolded protein can be corrected by pharmacological agents, such as miglustat [10], but their correcting properties appear to be less efficient in preliminary clinical trials. CF is also associated with an altered lipid homeostasis, with a decrease of PUFA in CF tissues and plasma [15]–[19]. Nevertheless, the SFA compositions of membrane PL, which have been shown to modulate crucial biophysical properties in several cellular cell types and to regulate several steps of the secretory pathway [23] and could therefore alter F508del-CFTR trafficking correction by pharmacological means, had not been studied so far.

In the present study, we show that a Palmitate accumulation occurs within CF patient bronchial epithelial cells with, as a corollary, an increase of the PC saturation rates. This accumulation is not observed in the corresponding CFBE41o^−^ cell line under standard culture conditions (Fig. 1). We formulated the hypothesis that Palmitate accumulation could be the consequence of hypoxia, which relates to the thick layer of mucus obstructing the lungs [30], because fatty acid desaturases are oxygen-dependent [31]. Thus, we tried to culture the CFBE41o^−^ cells in a hypoxia chamber (Fig. 2). This led to a significant increase of the PC saturation rate, but Palmitate accumulation remained too limited in these conditions to mimic the PC pattern observed in CF freshly isolated cells. Since the chronic hypoxia encountered in CF patients, and probably responsible for the Palmitate accumulation within PC, appeared to be too difficult to achieve using this method, we developed an alternative strategy consisting in feeding the cells with an exogenous Palmitate source, as already described for β cells [25]. Among the various Palmitate concentrations tested, incubation of CFBE41o^−^ cells with 100 µM Palmitate resulted in a PC profile that appeared fairly similar to the one encountered with CF patient (Fig. 3). Interestingly, if higher Palmitate concentrations resulted in apoptosis induction (250 µM Palmitate; Fig. 4), 100 µM Palmitate did not appear obviously toxic to the cells. A closer examination revealed that incubation of CFBE41o^−^ cells with 100 µM Palmitate resulted in an increased phosphorylation rate of eIF2α, a known marker of the SFA-induced ER stress [25], [35], [37], [38]. Altogether, these data suggest, if extrapolated to the epithelial airway cells from CF patients, that those cells display a lipointoxication-like phenotype.

UPR induction in CF is a controversial subject. Some studies demonstrated an atypical UPR induction in CF, characterized by the lack of the PERK-eIF2α induction [41], [42], while others showed the absence of ER stress in CF cells [43]. These discrepancies could result from the use of very different cellular models. We therefore propose, based on the results obtained in this study with CFBE41o^−^ cells improved to mimic the PC pattern observed in freshly isolated CF cells (Fig. 4B), that UPR induction is not related to *F508del-CFTR* expression *per se*, but to secondary alterations in the cellular lipid composition (lipointoxication) related to hypoxia.

A very interesting point is the role of Palmitate in β-cell death and its relevance to the etiology of Type 2 diabetes. As already mentioned, such as demonstrated here for epithelial cells, β-cell exposure to long chain SFA, including Palmitate, results in ER stress [35], [37], which can ultimately leads to cell death by apoptosis. This process is believed to contribute to the decrease of the β cell mass, leading to a deficient insulin secretion and therefore the development of Type 2 diabetes [44]. In CF, with the advancing patient age, the most common comorbidity is cystic fibrosis-related diabetes (CFRD) [45]. It has been shown in CF β-cells an abnormal Cl^−^ channel function leading to thick viscous secretions obstructing the exocrine pancreas [46], [47], and a significantly reduced percentage of insulin-producing cells within islets [48]–[51]. The data presented in this study may shed a new light on the underlying mechanisms. Indeed, one may assume that hypoxia-driven Palmitate accumulation may well be a crucial determinant for β-cell dysfunction in CFRD. Further characterization of PC composition in pancreatic cells from CF individuals will undoubtfully bring new insights in the comprehension of CFRD etiology.

In previous studies, it has been shown that Ca^2+^ homeostasis is altered in CF cells compared to non-CF cells, with a decrease of global Ca^2+^ mobilization [52], [53], as a result of F508del-CFTR retention in the ER [54]. It has also been shown that ER Ca^2+^ stores are not different in both CF and non-CF cells [53]. Our data are consistent with these previous results and show that Palmitate accumulation has additive effects on calcium homeostasis disruption in CF cells.

We have also shown that increasing the saturation rate of PC has a significant impact on F508del-CFTR trafficking correction by miglustat and isoLAB. Indeed, the incubation of corrected CFBE41o^−^ cells with 100 µM Palmitate results in the decrease of F508del-CFTR iodide efflux, reflecting the Cl^−^ current. To account for this observation, we can formulate two hypotheses, which may be not exclusive one to the other: the decrease in Cl^−^ current could be related to alterations in trafficking correction of the F508del-CFTR protein to the plasma membrane due to general impacts of saturated-PL on the secretory pathway, or to a loss of function of the corrected protein. High saturated-PL levels impact membrane properties in many ways. First, they tend to decrease membrane fluidity/increase membrane order, which is not compatible with most of the cellular processes that require high membrane dynamics, such as protein translocation/folding in the ER [21]. Second, they tend to increase bilayer thickness. Thickness of the hydrophobic membrane-spanning regions of an integral protein should match the thickness of the bilayer to avoid exposure of hydrophobic residues to water, a phenomenon known as hydrophobic-mismatch (for review, see [55]). As a consequence, increasing membrane thickness may have dramatic consequences on the folding, and therefore the trafficking and/or function, of membrane-embedded proteins such as the CFTR channel. Finally, PL shape itself modulates important biophysical parameters, including membrane curvature and lipid packing [23]. As shown on Fig. 1E, PC species containing two SFA display a cylindrical shape (DB = 0, Fig. 1E) while PC containing two UFA display an overall conical shape (DB = 2, Fig. 1E). Therefore, Palmitate accumulation within PC species is expected to shift the shape of the overall PC population from conical to rather cylindrical (Fig. 1E). Conical lipids are required to form areas of positive and negative curvature, and therefore SFA-related conical-PL depletion is expected to alter membrane bending. Moreover, conical lipid deprivation also results in increased lipid packing [23] which alters the recruitment of loose lipid packing-sensing proteins required for optimal budding, to nascent vesicles. Altogether, these processes could account for a global disruption of the entire secretory pathway.

Overall, SFA accumulation within membrane PL is expected to have very broad and general impacts on plasma membrane-embedded proteins in the CF cell. In fact, in this study, we measured the activity of three Cl^−^ channels localized at the plasma membrane, namely Cl_Ca_, Cl_swell_ and the corrected F508del-CFTR after incubation of CFBE41o^−^ cells with 100 µM Palmitate. Even if these three Cl^−^ channels are differently activated (Cl_Ca_ by calcium, Cl_swell_ by cell swelling, and corrected F508del-CFTR by phosphorylation and ATP hydrolysis), we could relevantly show that these Cl^−^ channels and plasma membrane Ca^2+^ influx are impacted either in their trafficking and/or function under SFA accumulation, with, as a consequence, a significant decrease in iodide efflux (Fig. 6) and calcium intake (Fig. 5B). This strongly suggests that bronchial epithelial cells are lipointoxicated by SFA in CF patients, a process that ought to be considered for the development of future therapeutic strategies.

To conclude, if understanding the precise impacts of SFA on F508del-CFTR trafficking and/or function will require further work, such as the analysis of the delivery of the mutant form to the plasma-membrane under SFA-accumulation in overexpressing models, data presented in this study may bring some new explanations concerning the discrepancies between *in vitro* and *in vivo* effects of correctors. We propose that hypoxia-related SFA accumulation impacts on membrane properties could be an important bottleneck for F508del-CFTR trafficking correction by pharmacological means in clinical trials. Relieving lipointoxication in CF cells may well be a way to synergize corrector effects and optimize their therapeutic action.

## Supporting Information

File S1 Table S1, PC composition of CF and non-CF cells. Table S2, PC composition of 16HBE14o^−^ and CFBE41o^−^ cells after incubation in a hypoxia chamber. Table S3, PC composition of CFBE41o^−^ cells after incubation with different palmitate concentrations.
(DOCX)
