# Supplementary material for: Cystic Fibrosis Bronchial Epithelial Cells Are Lipointoxicated by Membrane Palmitate Accumulation
Source: PLoS One. 2014 Feb 19;9(2):e89044. doi: 10.1371/journal.pone.0089044 (PMC3929646; doi:10.1371/journal.pone.0089044)
Supplement: File S1 — Table S1, PC composition of CF and non-CF cells. Table S2, PC composition of 16HBE14o− and CFBE41o− cells after incubation in a hypoxia chamber. Table S3, PC composition of CFBE41o− cells after incubation with different palmitate concentrations. (DOCX) [file pone.0089044.s001.docx]

**Table S1: PC composition of CF and non-CF cells**

Results are percentage of PC means ± SD, obtained from the mass spectra of at least three independent experiments.

| PC | (30:0) | (30:1) | (32:0) | (32:1) | (34:0) | (34:1) | (34:2) | (36:1) | (36:2) | (36:3) | (38:2) | (38:3) |
| --- | --- | --- | --- | --- | --- | --- | --- | --- | --- | --- | --- | --- |
| m/z [M+H]^+^ | 706 | 704 | 734 | 732 | 762 | 760 | 758 | 788 | 786 | 784 | 814 | 812 |
| Non-CF patients | 0.7  ± 1.1 | 1.8  ± 1.7 | 8.5  ± 0.8 | 2.7  ± 2.4 | 4.1  ± 1.2 | 23.8  ± 8.0 | 15.8  ± 7.1 | 8.8  ± 3.3 | 15.9  ± 2.1 | 8.8  ± 3.0 | 2.8  ± 0.8 | 6.4  ± 2.6 |
| CF patients | 1.80  ± 2.2 | 3.39  ± 1.4 | 18.37  ± 3.3 | 4.48  ± 1.6 | 4.31  ± 0.2 | 24.14  ± 2.1 | 10.94  ± 1.4 | 6.10  ± 2.5 | 10.27  ± 2.1 | 8.23  ± 3.5 | 2.04  ± 1.4 | 5.92  ± 2.1 |
| 16HBE14o^-^ | 0  ± 0 | 0  ± 0 | 3.1  ± 0.8 | 0.9  ± 1.3 | 1.2  ± 1.7 | 13.6  ± 1.5 | 24.5  ± 2.1 | 11.1  ± 1.6 | 38.2  ± 3.2 | 4.9  ± 0.02 | 0  ± 0 | 2.5  ± 0.05 |
| CFBE41o^-^ | 0  ± 0 | 0  ± 0 | 1.6  ± 2.3 | 0.9  ± 1.3 | 1.1  ± 1.5 | 13.0  ± 2.0 | 25.8  ± 2.0 | 10.8  ± 1.5 | 40.0  ± 6.5 | 5.2  ± 0.7 | 0  ± 0 | 1.6  ± 2.2 |

**Table S2: PC composition of 16HBE14o^-^ and CFBE41o^-^ cells after incubation in a hypoxia chamber**

Results are percentage of PC means ± SD, obtained from the mass spectra of three independent experiments.

| PC | (30:0) | (30:1) | (32:0) | (32:1) | (34:0) | (34:1) | (34:2) | (36:1) | (36:2) | (36:3) | (38:2) | (38:3) |
| --- | --- | --- | --- | --- | --- | --- | --- | --- | --- | --- | --- | --- |
| m/z [M+H]^+^ | 706 | 704 | 734 | 732 | 762 | 760 | 758 | 788 | 786 | 784 | 814 | 812 |
| 16HBE14o^-^  hypoxia | 1.2  ± 1.6 | 2.4  ± 0.3 | 4.9  ± 1.6 | 2.5  ± 0.5 | 3.2  ± 0.3 | 18.7  ± 2.5 | 22.9  ± 2.1 | 9.4  ± 2.0 | 25.4  ± 3.1 | 5.6  ± 1.1 | 0.9  ± 1.3 | 3.0  ± 1.4 |
| CFBE41o^-^  hypoxia | 1.3  ± 1.8 | 2.8  ± 0.4 | 4.9  ± 0.6 | 2.6  ± 0.04 | 3.4  ± 0.8 | 17.3  ± 0.3 | 22.1  ± 3.3 | 10.2  ± 1.4 | 26.1  ± 2.5 | 6.1  ± 1.3 | 0.7  ± 1.0 | 2.5  ± 0.6 |

**Table S3: PC composition of CFBE41o^-^ cells after incubation with different palmitate concentrations**

Results are percentage of PC means ± SD, obtained from the mass spectra of at least three independent experiments.

| PC | (30:0) | (30:1) | (32:0) | (32:1) | (34:0) | (34:1) | (34:2) | (36:1) | (36:2) | (36:3) | (38:2) | (38:3) |
| --- | --- | --- | --- | --- | --- | --- | --- | --- | --- | --- | --- | --- |
| m/z [M+H]^+^ | 706 | 704 | 734 | 732 | 762 | 760 | 758 | 788 | 786 | 784 | 814 | 812 |
| CFBE41o^-^  BSA | 1.3  ± 1.9 | 1.0  ± 1.4 | 6.1  ± 1.5 | 3.9  ± 0.6 | 3.7  ± 0.4 | 26.3  ± 4.1 | 16.6  ± 2.2 | 7.8  ± 0.02 | 15.2  ± 2.2 | 9.7  ± 3.2 | 2.0  ± 0.1 | 4.2  ± 0.7 |
| CFBE41o^-^  10 µM Pal | 1.0  ± 1.4 | 0  ± 0 | 13.5  ± 4.1 | 4.9  ± 0.3 | 4.6  ± 0.5 | 29.4  ± 2.1 | 15.1  ± 1.0 | 6.9  ± 0.1 | 11.7  ± 2.7 | 7.7  ± 1.6 | 0.9  ± 1.3 | 2.9  ± 0.2 |
| CFBE41o^-^  25 µM Pal | 0.8  ± 1.2 | 0  ± 0 | 10.5  ± 0.4 | 5.8  ± 0.1 | 4.7  ± 0.5 | 33.6  ± 1.2 | 18.9  ± 1.4 | 5.8  ± 0.1 | 10.3  ± 0.5 | 6.4  ± 0.4 | 0  ± 0 | 2.2  ± 0.1 |
| CFBE41o^-^  50 µM Pal | 0  ± 0 | 0  ± 0 | 13.6  ± 1.9 | 7.0  ± 0.1 | 4.7  ± 0.1 | 30.8  ± 2.2 | 20.5  ± 1.2 | 6.3  ± 0.1 | 10.4  ± 0.5 | 4.4  ± 0.8 | 0  ± 0 | 2.3  ± 0.2 |
| CFBE41o^-^  80 µM Pal | 0  ± 0 | 0  ± 0 | 14.6  ± 0.9 | 8.2  ± 0.03 | 4.8  ± 0.2 | 33.0  ± 0.4 | 20.3  ± 0.6 | 5.3  ± 0.3 | 8.6  ± 0.5 | 4.3  ± 0.6 | 0  ± 0 | 0  ± 0 |
| CFBE41o^-^  100 µM Pal | 0  ± 0 | 0  ± 0 | 15.8  ± 2.6 | 8.7  ± 0.4 | 4.6  ± 0.4 | 31.1  ± 0.9 | 21.7  ± 0.7 | 4.9  ± 0.02 | 8.6  ± 1.0 | 4.0  ± 0.3 | 0  ± 0 | 0.6  ± 1.0 |
| CFBE41o^-^  150 µM Pal | 1.0  ± 1.3 | 0  ± 0 | 37.2  ± 9.6 | 5.0  ± 2.2 | 4.1  ± 0.1 | 16.7  ± 4.1 | 13.4  ± 2.0 | 2.8  ± 0.6 | 4.5  ± 0.6 | 13.0  ± 1.3 | 0  ± 0 | 2.4  ± 0.1 |
| CFBE41o^-^  250 µM Pal | 0  ± 0 | 0  ± 0 | 52.4  ± 1.3 | 7.0  ± 0.1 | 3.5  ± 0.4 | 16.9  ± 1.0 | 13.7  ± 0.5 | 0  ± 0 | 3.5  ± 0.1 | 3.0  ± 0.04 | 0  ± 0 | 0  ± 0 |
| CFBE41o^-^  500 µM Pal | 0  ± 0 | 0  ± 0 | 82.4  ± 1.8 | 0  ± 0 | 0  ± 0 | 10.0  ± 1.0 | 7.6  ± 0.8 | 0  ± 0 | 0  ± 0 | 0  ± 0 | 0  ± 0 | 0  ± 0 |
| CFBE41o^-^  1 mM Pal | 0  ± 0 | 0  ± 0 | 77.7  ± 13.4 | 0.8  ± 1.6 | 0  ± 0 | 9.9  ± 2.1 | 7.8  ± 2.9 | 0  ± 0 | 0  ± 0 | 3.8  ± 7.5 | 0  ± 0 | 0  ± 0 |
